# How do Yolŋu recognise and understand their children’s learning? Nhaltjan ŋuli ga Yolŋuy nhäma ga märr-dharaŋan djamarrkuḻiw marŋgithinyawuy?

**DOI:** 10.1371/journal.pone.0272455

**Published:** 2022-08-18

**Authors:** Emily Armstrong, Ḻäwurrpa Maypilama, Lyn Fasoli, Abbey Guyula, Megan Yunupiŋu, Jane Garrutju, Rosemary Gundjarranbuy, Dorothy Gapany, Jenine Godwin-Thompson, Anne Lowell

**Affiliations:** 1 Northern Institute, College of Indigenous Futures, Education and the Arts, Charles Darwin University, Darwin, Northern Territory, Australia; 2 Yalu Marŋgithinyaraw Indigenous Corporation, Galiwin’ku, Elcho Island, Northern Territory, Australia; 3 SNAICC—National Voice for our Children, Collingwood, Victoria, Australia; University of Queensland, AUSTRALIA

## Abstract

Indigenous families have culturally-specific strengths, priorities, and methods for assessing their children’s development. Recognition and support of children’s and families’ strengths are important for identity, health and wellbeing. However, strengths can be missed in assessment processes developed in non-Indigenous contexts. Yolŋu are First Nations Australian peoples from North-East Arnhem Land. This study was conducted to explore Yolŋu early childhood development, assessment and support in response to concerns that Yolŋu strengths and priorities are often not recognised. The cultural and linguistic expertise of Yolŋu researchers was central in this qualitative study. Rich empirical data were collected through a form of video reflexive ethnography with six children and their extended families over seven years and through in-depth interviews with 38 other community members. An iterative process of data collection and analysis engaged Yolŋu families and researchers in a collaborative, culturally responsive research process which drew on constructivist grounded theory methods. Findings illustrate how Yolŋu children are immersed in complex layers of intertwined and continuous testing and teaching processes integrating holistic frameworks of cultural identity and connection, knowledge and practices. Yolŋu families monitor and recognise a child’s development through both direct and explicit testing and through observing children closely so that children can be supported to keep learning and growing into their knowledge, strengths and identity. Yolŋu expressed concern that such learning is invisible when the child is viewed through non-Yolŋu lenses and assessed with processes and tools from outside the community. Indigenous peoples have a right to culturally congruent assessment of their children. Those who share the child’s culture and language have the expertise to ensure that cultural strengths and priorities are recognised and understood.

## Explanation of terms

*Yolŋu* are First Nations Australians from North-East Arnhem Land in the Northern Territory of Australia.

*Balanda* is a term used by speakers of Yolŋu languages to refer to non-Indigenous people.

*First Nations Australians* is used in this article to include diverse Aboriginal and Torres Strait Islander peoples in Australia. This term recognises the identities of First Nations peoples who hold unceded sovereignty over their lands and waters.

*Indigenous* is used in this article in reference to Indigenous peoples internationally or when this is the term used in the work of other authors referred to.

## Introduction

Indigenous families and communities around the world have rights and responsibilities to raise and educate their children in their own languages and according to their own cultural methods [1]. This includes Indigenous peoples’ rights to determine their own criteria for assessment of their children [2]. Upholding these rights requires recognition of the “fundamental heterogeneity” [3 p424] of children’s development and the culturally constructed nature of childhood developmental assessment processes [4]. This article explores knowledge and practices related to assessment of children in one cultural context–a very remote First Nations community in Northern Australia–drawing on findings from a study of Yolŋu child development and child-rearing [5].

The early years of children’s lives are a vital and rapid time of development when a child’s relationships and experiences shape their identity, learning, health and behaviour [6]. Each culture, and each family within a cultural group, has its own specific ways of raising children and interpreting and supporting their development [7, 8]. The lenses through which children are understood and supported are central to developing a strong identity and spirit and passing on cultural knowledge and practices through the generations [9].

We all see and understand the world, ourselves and other people through our own cultural lenses despite the common misperception in dominant colonial societies that culture is something that ‘other’ people have and that people from white, Western societies do not [10]. It is impossible to understand child development or child-rearing in a “cultural vacuum” [7 p293]. Ignoring cultural lenses (our own or other peoples’) risks misunderstanding each other, reduces the potential benefit of supports, “devalues the validity of other ways of knowing and continues to reinforce systemic discrimination and racism” [7 p293].

Assessment processes embed cultural philosophies and theoretical perspectives about how people develop and learn, and how development and learning can be monitored and measured [4, 11]. In this article, we will use the term ‘assessment’ broadly to mean any process that describes, measures, monitors, rates and/or compares children’s and family’s skills, resources, strengths, needs and goals for support. This broad conceptualisation of assessment recognises that many western-trained assessors aim to work in partnership with families and are informed by strength-based, family-centred models of practice [12–14].

In First Nations Australian communities, Balanda (non-Indigenous) controlled institutions have introduced various child assessment processes (for examples of assessment processes used in remote First Nations Australian communities see [15–17]). Early childhood assessment processes commonly used in health and education services are primarily informed by developmental theories of child development [18] and hold power in Australian communities: they are used by institutions and organisations to identify children’s strengths and needs and to determine how funding is spent on interventions and support. Some models of assessment have been critiqued as applying European middle-class norms as if they were universal measures of a linear sequence of child developmental milestones [4].

Even within non-Indigenous contexts, there are alternative theories and approaches to children’s learning and assessment [18, 19]; it can not be assumed that institutional ways of assessing children align with the values and cultures of all families [20]. For Indigenous peoples living in settler colonised countries, the cultural norms, philosophies, theories, values and frameworks applied in commonly used early childhood assessments may conflict with or sit outside families’ own child-rearing knowledges and practices [21, 22], overlooking family and community “values and concepts of development” [23 p3]. Many Indigenous academics have asserted that only a person from the same culture as the child can really “see” the child, for example:

“European assessment is totally different than Māori assessment because our eyes look at it different…. you have to see it through Māori eyes in order to understand.”

[24 p246]

In Australia, there is evidence and community concern that commonly used assessment processes and tools have failed to recognise First Nations Australian children’s differences and strengths and have instead foregrounded needs and deficits [5, 25–28]. When non-Indigenous early childhood practitioners work through their own lenses, many “appear unable to see the strengths that Aboriginal children bring” [25, p90]. National and international assessment procedures have been described as culturally biased towards Western knowledges and practices, perpetuating marginalisation of children and families from non-Western backgrounds and marginalisation of Indigenous knowledges and practices [29]. Using childhood assessment tools with a population for whom they were not designed can lead to inaccurate and incomplete assessment of skills [30–32] and stereotyping of Indigenous children as less capable [21, 23].

Imposing the socio-cultural assumptions of one culture on the children of another culture has been described as: undermining [33], inaccurate, biased, invalid, harmful [34], problematic, disempowering and incoherent [4]. For example, Simpson, a Michi Saagiig Nishnaabeg scholar, writer and artist, described her own education experience in Canada as a non-consensual process “of being continually measured against a set of principles that required surrender to an assimilative colonial agenda” [35 p6]. Assessment tools represent and normalise the values, goals and practices of the organisations who create the tools [36]. The underlying values and goals of assessments may not align with Indigenous peoples’ own definitions of success for their children [33, 37] and may be incongruent with community priorities for holistic, culturally sensitive, family-centred support for children [23].

Research and systemic change are required to 1) respect, recognise, learn from and support the strengths of the ways Indigenous peoples raise children [22, 33]; and 2) challenge assumptions embedded in existing culturally mismatched paradigms for assessment of Indigenous children’s development [4, 30]. Such changes have the potential to impact positively upon the identity, health and wellbeing of children, families and communities [38].

Reconceptualising assessment of Indigenous children requires recognition of the “taken-for-granted views” [39 p17] embedded in early childhood assessment processes–it requires researchers, policy makers and practitioners to ask:

“‘whose values and beliefs are being centred and privileged?’; ‘whose values and beliefs are made invisible?’”

[39 p17]

Reconceptualising early childhood assessment also requires the centring of the knowledges, practices, scholarship, terms of reference and agency of Indigenous peoples [39]. As Jane Garrutju, a Senior Yolŋu woman from North-East Arnhem Land in Australia said:

“Don’t drag us to your world because we don’t know how you grow *your* children, you don’t know how we grow *our* children. … We are not robots and you’re switching us and telling us to do this and do that. We are people with brains, people with vision and dreams, we are people with dignity”

[40].

In response to such concerns, the aim of this article is to share research findings to promote recognition of Yolŋu knowledges and practices of monitoring and supporting children’s development. Yolŋu are First Nations Australian people from North-East Arnhem Land in the Northern Territory where local knowledges, practices and languages are strong [41]. The research team and Yolŋu participants acknowledge the diversity within and across First Nations Australian communities: the findings from this study are intended to stimulate critical reflection but only First Nations Australians can assess their relevance to their own cultural contexts.

## Methods

### Research context

Our research was conducted in a very remote coastal community in North-East Arnhem Land in Northern Australia where 94% of the population identify as Aboriginal and/or Torres Strait Islander and less than 5% of people speak English at home [42]. This community is owned and controlled by Yolŋu traditional custodians of the land and surrounding seas. It is classified as very remote on the basis of relative access to services [43], many of which are managed from Darwin which is over 500km away by air.

In North-East Arnhem Land, Yolŋu children grow up in complex language environments where multiple traditional languages are spoken across generations [44] and Yolŋu sign language is also commonly used in everyday interactions [30, 44]. Children listen to English on digital media and in songs. English is used for interactions with people who are not Yolŋu, for example in early childhood programs, at the school and health clinic [5]. Children who attend health, education, child care and/or family support services are routinely assessed by multiple service providers using a wide variety of assessment tools and processes.

### Research design

The *Ŋuthanmaram djamarrkuḻiny’ märrma’kurr romgurr—Growing up children in two worlds* project (2013–2019) was a qualitative study, collaboratively designed with a group of senior Yolŋu women who were concerned that early childhood programs coming into their community did not recognise Yolŋu knowledge, strengths and priorities [9]. The research was guided and overseen by this group of senior women and other interested community members who formed the project’s *Ŋaraka-Ḏälkunhamirr Mala* (Backbone Committee).

Our research team included researchers from Charles Darwin University, Yalu Marŋgithinyaraw Indigenous Corporation and SNAICC: National Voice for our Children [45]. Yolŋu researchers and members of the Backbone Committee share the language and cultural backgrounds of the participating families and are connected through *gurruṯu* (kinship systems) to all Yolŋu in the community. Their cultural and linguistic knowledge and expertise were foundational to supporting culturally responsive processes throughout all stages of the study.

Our research approach was informed by our extensive, collaborative, community-based research in this context building on pre-existing relationships and connections—e.g. [25, 46–50]. The study design intentionally centred Yolŋu voices and control in the generation, collection, interpretation and dissemination of knowledge about Yolŋu children and families [5]. Using video reflexive ethnography [46, 49, 51], families and researchers collected and analysed videos of children’s learning in everyday home contexts. Data collection and analysis continued over seven years because participants wanted to share an abundance of evidence about their children to promote recognition of their practices and priorities. We used a collaborative and iterative process of data collection and data analysis, integrating elements of constructivist grounded theory [52] to construct concepts and theory based on participants’ own interpretations of the data.

### Ethics

The study received approval from the Charles Darwin University Human Research Ethics Committee (Phase 1 2013–15 H16025; Phase 2 2016–18: H12136) and from the Regional Council Local Authority representing local community members. Yolŋu ways of assessing children are Yolŋu intellectual property and will always remain so. The research findings included in this article have been shared through the collaborative authorship of Yolŋu and non-Yolŋu researchers and with the written consent of participants. Research findings are being shared according to community priorities through a variety of mediums including journal articles, community and conference presentations, a project website and online talking books (http://www.growingupyolngu.com.au).

Participation was voluntary and use of participants’ preferred languages in all stages of the research supported genuinely informed consent, centred Yolŋu knowledges, and supported meaningful data collection, analysis and dissemination. In disseminating the research findings, children are referred to using their *mälk* (skin names) which are shared by hundreds of other Yolŋu and so do not identify individuals. Children and adult participants have given their informed consent for use of data examples (e.g. video excerpts, audio recordings, written quotes) in specific dissemination activities and participants chose how this data should be attributed.

### Participants

Under the guidance of senior Yolŋu researchers and advisors, Yolŋu families were invited to participate. Some participants requested to join the study after hearing about it from other participants or researchers. Purposive sampling aimed to achieve diversity across family, clan and language groups (for details see [45]). Six children (three boys, three girls; aged between 2 months and 2 years old at commencement) and their extended family members (parents, grandparents, great-grandparents, siblings, aunts, uncles, cousins and others connected in the Yolŋu kinship system) participated in longitudinal data collection over seven years. Thirty-eight additional community members (aged 18–78) participated in in-depth interviews and fifteen of these families also requested video recording of their children.

### Data collection

Data were collected through a form of video-reflexive ethnography [46, 51] over seven years. More than 250 episodes of video data (approximately 100 hours in total) were recorded of children and their extended families engaged in everyday activities in a Yolŋu community, for example hunting/ gathering food and sharing it with family, dancing at *buŋgul* (ceremonies), playing and practicing skills at home, interacting and storytelling in extended family groups. Families and children had agency in choosing the time and place for data collection. Videos were recorded approximately every 3–6 months. Family members and Yolŋu researchers later participated in collaborative video analysis by reviewing the video recordings to identify and reflect on what children were learning and how this learning was supported and assessed.

The children’s key caregivers also shared their perspectives through conversational interviews and informal discussions with researchers at various times over the period of the study to suit participant preferences. Interview content was guided by what participants wanted to share with researchers and typically included discussion of the child’s development, family context, current strengths and challenges from Yolŋu family members’ perspectives.

Cross-sectional data from other community members were collected through in-depth interviews exploring their perspectives, knowledge and practice of child development and child-rearing. Responding to participant requests to collect additional video data was part of the community-led approach of the research. Participants who requested video recording of their children also engaged in collaborative video analysis with researchers.

All collaborative video analyses, interviews and discussions were conducted in participants’ preferred languages (usually a Yolŋu language) and digitally recorded. These recordings were then interpreted by Yolŋu researchers using meaning-based interpretation [53] and transcribed in English.

### Data analysis

A collaborative and iterative process of data analysis was ongoing throughout this project and was simultaneous with data collection. Yolŋu and non-Indigenous researchers worked together to analyse data from all sources, i.e. data from video analyses, interviews and discussions with family members. We used an inductive approach drawing on constructivist grounded theory methods [52] and qualitative data management software, *QSR Nvivo12* [54], to assist with data management and organisation.

First, data were grouped using initial codes derived from participants own words, some in Yolŋu languages and some in English. Researchers then worked collaboratively to group codes into conceptual categories using focused coding and a process of constant comparative analysis [52]. Emerging conceptual categories and the relationships between them were discussed amongst the research team, with Backbone Committee members and with participants to elaborate, refine and confirm emerging findings. Discussions amongst researchers and researcher analytic reflections were also recorded and transcribed as memos to provide synthesising interpretations of the data from perspectives of Yolŋu researchers who share the cultural background of participants. Key conceptual categories are shared in the findings section of this article marked with *‘inverted commas and italics’*.

## Findings

In this section we explore study findings related to Yolŋu ways of assessing their children’s development in early childhood that Yolŋu want others to recognise and respect. The concept of ‘*developing a strong Yolŋu identity’* was identified as the highest priority in early childhood for Yolŋu families. Yolŋu children’s identities are established through cultural connections from conception; Yolŋu families monitor how a child shows their identity and connections as they develop. An understanding of the child is built over time through relationships and close observation in everyday family and cultural contexts. Collection of data capturing interactions between families and children from infancy to 6–7 years old demonstrated how extended families monitor and support Yolŋu children through deepening relationships and expanding connections as children’s identities and skills develop.

One of the key ways in which extended families nurture and support Yolŋu children to develop their identities is by ‘*everybody testing and teaching all the time’*. Yolŋu families use testing and teaching processes which are intertwined rather than discrete. Processes vary depending on the child’s development and the skills and knowledge being learned. Yolŋu focus intensively on developing the child’s knowledge of their own identity and cultural connections to people, places, animals, plants and elements of the natural world. This knowledge is assessed and developed through ‘*testing them straight’*: direct and explicit testing and repetitive teaching using words and actions. As Yolŋu children grow and learn, their families expand this knowledge and closely monitor their development by ‘*walking beside them’*: watching them closely in everyday experiences and interpreting their actions.

For Yolŋu participants and researchers, the purposes of assessment are to ‘*recognise the child and our teaching’* so that children can be supported to keep learning and growing into their knowledge, strengths and identity. Individual differences are expected and accepted and are often attributed to the child’s family connections and environment. There are significant ‘*differences from Balanda’* which Balanda (non-Indigenous people) need to be aware of when working with Yolŋu children and families. During data analysis, Yolŋu researchers emphasised that children are ‘*showing Yolŋu skills that Balanda don’t see’*.

### *Waŋganyŋur*—all in one: Testing and teaching all the time

The process of assessing what a Yolŋu child knows is consistently held in connection with teaching–the two were not separated by Yolŋu participants and researchers. Yolŋu families test and teach children all the time and everywhere–“watching to see if he knows” for example, at home, out hunting, at *buŋgul* (ceremonies), around the community, at school. There is not a specific time, context and activity set aside for testing.

We don’t have markers to tick, what levels to tick. We just observe. It’s like our checklist in our head, you know? And we check in our memories.… We don’t give the time or date when to observe. We observe them when they are little, right up.

(Yolŋu Researcher)

Through this process Yolŋu are *‘learning from the child’*:

… how they respond on what we were telling them… I learned from that, I watched her.… I learned from my granddaughter Ŋarritjan, how she was getting the understanding…. by us telling her, she’s not learning on her own.

(Yolŋu community member)

Yolŋu researchers emphasised that the reason for testing is *not* to divide children into categories or determine who is the smartest but to recognise their strengths and identify when more support is needed:

We don’t test to divide them into smart, middle and last. No, we just want to see what they know and if they’re learning. Why? So we can try to develop them, give them more support. We’re building up their brain every day and the brain gets stronger through talk every day, developing and thinking.

(Ḻäwurrpa Maypilama, Yolŋu Researcher)

Processes of testing and teaching are intertwined, continuous and enacted both through direct and explicit testing and through observing children closely to monitor and recognise a child’s skills and knowledge.

### When children are little we test them straight: Asssessing learning of *gurruṯu* (kinship connections)

Understanding *gurruṯu* (kinship connections) was the most frequently and directly tested area of knowledge development for Yolŋu children in this study. The Yolŋu *gurruṯu* system is a highly complex kinship system and “it has always been there in Yolŋu blood–it has not changed” (Ḻäwurrpa Maypilama, Senior Yolŋu Researcher). As they grow, children are expected to gradually learn and understand more complex aspects of the *gurruṯu* (kinship) system. *Gurruṯu* includes names and sign language for relationships with close family and extended family, what to call people, *bäpurru* (clans), *wäŋa* and *yirralka* (homelands and clan estates). Each person in the *gurruṯu* (kinship) system has a *mälk* (skin name) and a moiety (*Dhuwa* or *Yirritja*) which also connects them to other people, animals, plants and aspects of the environment–these are interwoven systems.

Babies and young children, from birth to around two to three years old, are routinely tested while they are learning kinship connections by “asking the child straight—not to see how smart they or who is the smartest, but to see how they’re learning so we can teach them more” (Dorothy Gapany, Yolŋu Researcher). *Gurruṯu* is central to Yolŋu children’s identity, relationships and interactions–and to understanding their responsibilities to look after each other and learn from one another.

The importance of *gurruṯu* (kinship) is demonstrated by the frequency and intensity with which it is tested and taught when children are very young. While children have agency over choosing other skills and knowledge to focus on, families intensively test and teach *gurruṯu* with *all* Yolŋu children.

I’m asking Baŋaḏi [6 months old] where his grandmother is and where his uncle is and seeing if he can understand what I am saying to him. And if he knows and recognises who I’m trying to get him to show me. … So first we teach babies how he’s related to each one of the families.

(Baŋaḏi’s *Märi*—Maternal grandmother)

Understanding of the *gurruṯu* system is the foundation on which children build wider knowledge and skills.

The most important part is learning his connections about *gurruṯu* (kinship) and building confidence to dance and to know the songlines and to know where he’s standing between the songlines and the people who he’s dancing with.

(Gudjuk’s *Ŋäṉḏi—*Mother)

Yolŋu test infants’ understanding of *gurruṯu* (kinship) connections by noticing babies’ emotions and how they are “sensing people” around them. The baby should feel comfortable and happy if their close family is nearby and this is a sign that they already recognise their family. When family members visit a baby, they immediately introduce themselves using *gurruṯu* terms and Yolŋu sign language then observe how the baby responds. As children grow, Yolŋu adults or older children ask questions or do something to test how the child “responds back”.

Family watch carefully because the baby might respond using facial expressions (e.g. smiling), eye gaze (e.g. looking at the right person), actions (e.g. pointing, sign language, moving to the person), or making sounds or talking. These responses show whether the child recognises people and knows how they are connected through *gurruṯu*.

For example, when Gutjan was ten weeks old, her *Ŋathi* (maternal grandfather’s brother) introduced himself to her and tested her understanding and expression of *gurruṯu*. He held her up face-to-face with him and spoke with excitement and animation. He repeatedly stated his kinship connection “athi” (‘grandfather’ in the child language register—*yalŋgi matha*) and “ŋaya” (‘me’ in his own language, Galpu). Then he tested Gutjan by pausing for a response. He noticed when the baby smiled and when she made eye contact and looked at his mouth. He was excited when baby Gutjan made a sound and used intonation to talk to him. Gutjan said a sound which her *Ŋathi* (maternal grandfather’s brother) interpreted as ‘Yo!’ (‘Yes!’). He copied her sound back. These were baby Gutjan’s ways of showing that she recognised her *Ŋathi* by using her face, eyes and voice when her family tested her on *gurruṯu*. When Gutjan was a little older (7 months), she pointed and used Yolŋu sign to answer her family’s questions about her connections to people and totems.

If a child does not respond to testing of their knowledge of *gurruṯu* (kinship connections), it may be interpreted that he/she is confused and needs more teaching. For example, when Balaŋ was 9 months old his family expected him to use a sign to show that he was starting to learn the kinship system as it cycles over four generations. Their testing revealed that he did not know this yet and so his family showed him, pointed and said and signed *gurruṯu* terms over and over.

Families sometimes continue to use direct questions to test 3 or 4 year old children on more complex aspects of *gurruṯu* but this becomes less frequent as the child grows. For example, Gamarraŋ’s (4 years 6 months) father and cousin tested his knowledge of his clan connections with ceremonial dances by naming a group of people and seeing that he could do the dance associated with that group in response.

*Gurruṯu* (kinship connection) testing—teaching—testing—teaching routines continue until the child has demonstrated strong understanding of kinship concepts and their own relationships. Family members continue to monitor the child’s deepening knowledge of more complex elements of the kinship system primarily through observation of the child in everyday life rather than direct testing.

### As *djamarrkuḻi* (children) grow up we walk beside them and watch to see which knowledge that child caught

Yolŋu children demonstrate learning through their participation in everyday activities and interactions alongside older children and adults. For example, when Yolŋu watch children participating in ceremonial events, they are noticing how a child integrates and demonstrates the connections between many different aspects of learning. This is an opportunity for culturally specific, holistic assessment of knowledge as well as teaching to pass on knowledge.

But it has to be real life, not theory. It’s a practical thing that we’re teaching our children, how we were taught. … So when I see kids, strong, I straight away recognise that *yothu* (child). It’s from that family line. … And also that child is trying her best. … To keep that teaching–to grip that teaching! Grip on, hold onto it. Recognising yourself and who you are and show the other world about your knowledge … your outcome of Yolŋu way of learning.

(Jane Garrutju, Yolŋu Researcher)

For example, in a video recorded at a *buŋgul* (ceremony), two cousin-sisters danced the correct steps associated with their *wakupulu* (great-grandmother’s clan’s) songline. They demonstrated that they could listen and respond to the music and copy (at 3 years old) or remember (at 5 years old) the steps. Their mothers sat close behind them, encouraging them, giving them instructions about how they should dance and telling them about their connections to this ceremony and the clan who is leading it. Another video showed the same two girls dancing with the adult women two years later. A senior woman analysing this second video described it as a “test of connections to clan, songlines and dance”. Murimuri (at 7 years old) was dancing confidently and independently at the front of the group at her *Ŋathi*’s (maternal great-uncle’s) funeral. She was “respect dancing” for her *Ŋathi*. Her family and Yolŋu researchers chose these subtitles for this section of the video data (available online: https://youtu.be/SdzaiwZ6Crk):

We can see that Murimuri (now 7 years old) is dancing the way an adult dances in everything–the way she moves, her concentration, her connection to her *wakupulu manikay* (great-grandmother’s clan’s song). When the clapsticks start, she goes for it! This shows a test of her mind–of her connection to this group. Murimuri listens to how the songlines go and she responds. This video shows that Murimuri is capable and brave with dancing. She doesn’t have to ask people or wait for instructions. She just gets up and dances like an adult.

*Djamarrkuḻi* (children) are expected to follow their own interests and abilities by choosing which skills and knowledge they focus on and develop most strongly. Family members monitor Yolŋu children’s learning and development by observing and interpreting how each child engages and responds to experiences–for example how they communicate, how they act, their confidence and how they figure things out.

We just give them patterns of Yolŋu knowledge–we just throw that to the child. And that child will figure it out. They will learn. We watch and then we talk about it, so we know which knowledge that child caught. We just see for ourselves or we talk about that with family members. Like I would just see and think ‘Oh, that child he needs to ___ or he’s really strong on ___’

(Yolŋu Researcher)

Testing and teaching are constantly interwoven: if Yolŋu family notice a child is ready to learn a skill but seems to be finding it difficult, they may provide specific support or teaching for that skill. For example, when children are learning to walk, family watch closely how the child is standing up, balancing and taking their first steps. If a child is wobbly, struggling or slow to crawl or walk, the family might arrange hot sand treatment: heating sand under a small fire on the beach or in the bush-land then pressing that warm sand on the baby’s feet, legs, hips and spine “to make it warm and strong so she can walk by herself.” (Ḻäwurrpa Maypilama, Senior Yolŋu Researcher).

In this study, Yolŋu families consistently noticed small details in children’s behaviour which showed “which knowledge that child caught”. Yolŋu family analysis of video data demonstrated a type of continual observational assessment grounded in Yolŋu knowledge and relationships with their children.

My two year old *Ŋaṉḏi* (Mother), she understands the *gurrutu* (kinship) system and she put me in that layer. … and put it into words. That was amazing! She knows how everyone’s related to her and she’s building up that concept herself through her *dhukarr* (pathway). It’s in their *muḻkurr* (head/ knowledge), *rumbal* (body), *mel* (eyes), *goŋ dhu djäma* (actions of their hands). The practical comes out from what’s within them–what they can do to act upon what they’re thinking.

(Rosemary Gundjarranbuy, Yolŋu Researcher)

Yolŋu adults “walk beside” their children, including them as part of everything that is going on. Yolŋu children express their identity through showing what they know. The child has agency and leads the way, metaphorically and sometimes literally, and adults monitor the child’s interests and developing knowledge by following them.

Yolŋu adults notice what children notice. Children’s awareness of their environment, and responses to their environment, are important so Yolŋu notice and interpret the ways children respond to sounds and signs around them. For example, Baŋaḏi (4 years old) was playing at the edge of the water with another child and his dog when he noticed and mentioned the footprints of a crocodile. His grandmother commented on his important awareness of the crocodile footprints in the sand.

Yolŋu adults monitor children’s awareness of respectful behaviour and cultural conventions. For example, when a Yolŋu person passes away, their name is not spoken for some time, often several years, and similar sounding words and names are also avoided.

The kids always correct each other if they call each other the wrong kin or say a name like someone who passed away. The kids are really smart at it–they listen, even if you think they’re not, and if you say something that is like the name of someone who passed away, they will tell you off, not to say that name. We notice that, we listen for that.

(Yolŋu Researcher)

When older people hear a young person tell a story, they assess if the child has memorised it well–“if they have got all the story and kept it inside.” Some participants have described this as “pasting a picture in the child’s head” or seeing if the child is “photocopying”.

Even though the language was too deep, the child sang and cried the same words. She copied her *waku*’s (great-grandmother’s) song.

(Ḻäwurrpa Maypilama, Senior Yolŋu Researcher)

### Inside their bloodstream they’ve got that skill–We’re looking for them to show it

When Yolŋu monitor a child’s development, they are predicting and looking for strengths and skills that they expect the child to have because of the child’s family connections.

When we’re teaching children we straight away think of their ancestors–of their family line, fathers side and mothers side too.

(Jane Garrutju, Yolŋu Researcher)

Although there is a focus on strengths, family also recognise delays or weaknesses which may be in the family line and work together to support the child in these areas of learning. Children are accepted for who they are and family recognise their identities, personalities and abilities from a very young age because “inside his blood stream, he’s got that skill. We’re looking for it–for him to show it.” (Ḻäwurrpa Maypilama, Senior Yolŋu Researcher)

Because of their connections, you know what’s already there in the child, passed on from generation to generation. We look for that to come out in the child–she’s automatically formed because of her connections, her environment, how she grew up. And then she shows that. We can’t change it, it’s there. It’s in the foundation. We are testing which one she is learning strongly.

(Ḻäwurrpa Maypilama, Senior Yolŋu Researcher)

For example, Gutjan’s *Momu* (paternal grandmother) was good at hunting so her family expected to see these traits in Gutjan as she grew up:

Gutjan (aged 6) likes to go hunting. She likes to hunt for *maypal* (shellfish) and *ŋarirri’* (fish) and *nyoka* (mud crab). Her *Momu* (paternal grandmother) loved to go hunting every day and Gutjan has got that same skill for hunting–she inherited it from her grandmother who passed away before she was born. It’s in her family line.

(Gutjan’s *Ŋäṉḏi—*Mother)

Gudjuk’s family recognised that he had a strong interest in *buŋgul* (ceremonial dancing) and was a good dancer from a young age. His mother reported that:

He got that talent from his father… he used to take him along when there was a funeral or there was cultural events happening when he was a baby. And then whatever he was seeing on that age, while he was a baby, it all got stuck in his head while he was growing up.

(Gudjuk’s *Ŋäṉḏi—*Mother)

As a young child, Gudjuk often chose to join in at *buŋgul* (ceremonies) and sometimes he danced at ceremonies all day with his father, uncles and grandparents. When he was seven, Gudjuk’s mother described how she had monitored his development over time and identified how he was “learning to become a leader in the future”:

From when he was a baby–about 6 months old–Gudjuk started to learn his connections through songlines and *buŋgul* (ceremony). … His first words were copying the sounds that men make in the songs from his *waku* (great-grandmother) tribe like “brbrbr” and “Ga’y-ga’y! Ya’y-ya’y!”. When he started walking he started dancing. … Gudjuk has always been interested in dancing. Every time there is another tribe dancing, he picked up his *gaḻpu* (spear thrower) and … ran to join them. He likes using his *Ŋapipi*’s (Maternal uncle’s) *gaḻpu*. This is part of his learning how to become a leader in the future.

(Gudjuk’s *Ŋäṉḏi—*Mother)

As well as identifying and monitoring strengths, Yolŋu participants noticed when a child was “not strong on” a skill or area of development. They discussed this within the family and “accept it even though she is slow learning” while also providing active support to the child. Family worked together to offer more experiences and encourage the child to keep participating, keep trying and keep learning amongst the other children in the family.

For example, a grandmother and two aunts had noticed that Wamuttjan (2 years 8 months) was “talking in *yalŋgi matha* (baby language). Like she’s still a tiny *yothu* (baby or child)… but still we can understand”. Her family discussed that there were “three or four that aren’t strong in language” and that this appeared to be in the family line:

Same like her father–Wamuttjan is growing up exactly like her father. … They called him *ḻuku weṯi*—walking like a wallaby–but when he grew up his walking is fine. When he was growing up (in the city) his talking was exactly the same… and also thinking like Wamuttjan does—if you ask a question thinking then answering… responding slowly–but it’s normal. Also her father’s brother was the same–very late walking. (Wamuttjan’s *Mukul*—Paternal aunt)

Some of Wamuttjan’s family members commented that she was slower to learn to talk because she had been living with her mother in another community away from her extended family and therefore missing out on *‘testing and teaching all the time’* amongst many family members. So, while Wamuttjan was living in a different community, her *Momu* (paternal grandmother) rang her often to monitor her language development and encourage her:

With Wamuttjan, I was listening, she’s nearly getting there, nearly. I have been worried about her. That’s why when I ring (her mother), I say ‘give that phone to Wamuttjan so I will talk to her’.… To listen to her story, her sentence.

(Wamuttjan’s *Momu*—Paternal grandmother)

### Showing Yolŋu skills that Balanda (non-indigenous people) don’t see

During video analysis, Yolŋu families and researchers demonstrated detailed observation and interpretation of Yolŋu children’s understanding and skill development. Yolŋu adult expectations are responsive to each individual child and are based on close observation of the child’s developing skills paired with knowledge of a child’s identity, *gurruṯu* (kinship), family line and family context. Assessing development in this way requires deep Yolŋu knowledge and an ongoing relationship with the child and their family.

We’re teaching with a lot of layers and watching how they respond–which parts are they picking up? That is our testing.

(Ḻäwurrpa Maypilama, Senior Yolŋu Researcher)

As Yolŋu children pick up and show that they understand each layer of knowledge, their learning is celebrated by their extended family. However, these layers of learning are often invisible to those who come from different cultural and linguistic backgrounds to the child.

One case-study participant, Gutjan (3 years old), was also part of another research project. A Balanda (non-Indigenous person) travelled to the community to test Gutjan’s development. Although there were family members and a familiar Yolŋu teacher there as well, Gutjan (3 years) was silent with the stranger who was assessing her. Gutjan’s mother did not recall receiving any results from the assessment but was worried that the researchers would assume her child could not talk even though Gutjan’s extended family had noticed that language was one of her strengths. Her mother described the experience:

The Balanda (non-Indigenous person) brought books and blocks and activities and then we sat on the veranda with her … Gutjan was asked a question … what’s your *mälk* (skin name)? She’s not talking, maybe she’s too shy. Balanda was sitting there but normally she responds to the Yolŋu teacher. Balanda was sitting there trying to encourage her and then they were packing to leave. As soon as they got into the vehicle then Gutjan called out ‘Tata *Mukul* (kinship term, referring to Yolŋu Teacher)’… And then when the Balanda left, she was like a talking machine! ‘Give me a lolly. Cook me food. Bring me a drink–bring it to me, I’ll drink it!’ she was talking like that. ‘What kind of child are you?’ I said to her, ‘a talking machine?!’ Then I told that Balanda and at the same time she was writing it down. I told her she is a smart child and she loves to play and loves to talk.

(Gutjan’s *Ŋäṉḏi*—Mother)

Participants reported that when Yolŋu children get poor results on tests developed outside the community, it can lead to “Balanda thinking our children are not smart”.

The Balanda (non-Indigenous people) think we are ignorant. They learn in their own style and think they are smart and that we’re not smart…But we understand (the children) and when we speak to (Balanda), they don’t believe in us and take years and years in mocking us and seeing us as being black, without brains and don’t look after children. They don’t have compassion in the hearts of Balanda … They just see with their eyes but they don’t feel what our needs are. … You Balanda think like that about us, saying ‘what kind of people they are, they don’t look after their children’. We look after the children. We teach them the pathway … Get our word. Not just we get yours.

(Senior Community Member)

Yolŋu children demonstrate many complex and valuable skills which can remain invisible when the child is viewed through non-Yolŋu lenses. Yolŋu researchers and participants expressed concerns about the invisibility of Yolŋu skills when children are assessed with processes and tools from outside the community. Sharing the research findings in this article is intended to increase recognition of Yolŋu ways of assessing Yolŋu children and challenge the use of culturally mismatched assessment processes which embed Western-centric perspectives on child development.

## Discussion

Indigenous peoples’ ways of assessing Indigenous children have long been under-represented in the evidence base for early childhood assessment. To facilitate recognition and maintenance of Yolŋu ways of growing up children, Yolŋu participants in this study have shared extensive evidence of how their children develop knowledge and skills and how their development is monitored and supported by Yolŋu families.

Yolŋu assessors monitor and support Yolŋu children’s development using processes, contexts and expectations which are very different from the Western “taken-for-granted views” [39 p17] that have been introduced from the broader Australian context and embedded in common instutitional assessment tools and processes. Our research provides evidence of *what* is considered valuable and worth assessing from Yolŋu perspectives, and in *how and why* children’s developing knowledge and skills are tested. For these reasons, *who* is completing the assessment of a Yolŋu child is of critical importance. While Yolŋu children are growing up and living as part of two different knowledge systems (a Yolŋu system and a non-Indigenous system), this article focuses on Yolŋu expertise in monitoring and supporting their children’s learning that Yolŋu want others to recognise and respect.

### The importance of experienced, culturally connected assessors

Our findings demonstrate that Yolŋu children are assessed and supported in ongoing ways by both close and extended family members. Yolŋu children are also often assessed by non-Indigenous assessors who apply their specific skills and training, particularly from fields of Western education or Western health care. Many service providers who work with First Nations Australians do not share the culture, language, ideology and socialisation of the people with whom they work [30]. These assessors from different cultural backgrounds are likely to conduct and interpret their assessments very differently.

Our findings demonstrate the central importance of assessment of First Nations Australian children being conducted by or with those who: 1) are connected to the child and their family through relationships; 2) are walking beside and observing children during everyday experiences; and 3) have the cultural and linguistic knowledge to understand the layers and complexity of what and how children are learning. Many strengths of Yolŋu children, as illustrated in our findings, could be invisible or misinterpreted when assessed by a person from another cultural background using a culturally mismatched assessment process or tool.

Working interculturally is particularly challenging in First Nations Australian communities where differences between families and services providers in culture, language and power can be extensive [55]. Practitioners from outside the child’s cultural context need to work in culturally safe ways with local Indigenous partners by listening, interacting, practicing reflexivity and intentionally placing an Indigenous community’s vision in the centre of practice [30, 56]. Recognition of family members as experts is also relevant in non-Indigenous contexts, acknowledging the knowledge families hold of their child and context and the diversity within and between communities and families [57].

Non-Indigenous people working in partnership with First Nations Australian peoples have opportunities and responsibilities to challenge and transform current dominant systems of assessing Australian children so that First Nations Australian peoples’ ways of assessing their children are respected, valued, facilitated and maintained. Developing cultural responsivity in early childhood contexts requires non-Indigenous practitioners to stop enacting Western-based roles of evaluation, assessment, and intervention. Instead, non-Indigenous partners can: actively seek to partner with cultural experts [58, 59]; engage in reflexivity and examine cultural assumptions [10, 60, 61]; listen [62]; change practices to facilitate Indigenous peoples’ control over assessment processes [2, 34, 40]; and develop intercultural communication skills for engaging in this complex work [55, 63].

### The importance of culturally congruent assessment processes

The findings of this research demonstrate that Yolŋu interweave testing and teaching, assessment and support. Information gathered and shared by First Nations Australian assessors using culturally congruent assessment processes has the potential to reduce misinterpretation of difference as deficit and to increase wider recognition of children’s and families’ strengths and needs.

Yolŋu ways of testing children aim to recognise each child’s individual strengths and to support and develop each child’s learning. Yolŋu assessment is *not* a competitive process for testing who achieved set learning and who didn’t. This contrasts with cultural constructs of institutional teaching and assessment in contexts where children are tested individually then ranked in comparison with their peers. Our findings demonstrate that attempts to assess Yolŋu children at a set time, in a specific context or activity, can lead to the child not responding because they are “not ready” and family being concerned that others will think their children are “not smart”. Instead, Yolŋu children have agency in choosing which knowledge and skills they focus on and Yolŋu adults follow them, watching, noticing and talking about “which knowledge that child caught”. This way of assessing is familiar and comfortable for Yolŋu children and adults. It creates opportunities for holistic assessment of how a child integrates and practices the connection of many different aspects of learning within specific and valuable cultural practices alongside other children and adults. Such cultural difference in the process of assessment may make it inappropriate and difficult to adapt assessment models or tools from one culture to another.

Our study created opportunities for participants and researchers to analyse interactions between families and children over seven years to explore the depth and complexity of children’s skills and knowledge that are monitored and supported through relationships over time. The ways Yolŋu families assess children in everyday life are similar to the methodology used in this research: Yolŋu adults engaged in continual observation grounded in relationships with their children; then used Yolŋu languages and knowledges for collaborative, unstructured discussion and interpretation of what children’s behaviours showed about their identity, learning, development and support needs. These processes were facilitated by culturally responsive video analysis methods.

This process was considered culturally safe and responsive as videos were recorded by families and children themselves in everyday activities of their choice. In fact, participants requested more videos be recorded and analysed than were originally planned in the study. There were no pre-set expectations for what should be recorded or discussed as Yolŋu adult expectations were responsive to each individual child, their context and their family line. Yolŋu discussed children’s skills and needs within family groups rather than working alone to assess children’s development.

Application of a similar methodology may be considered by practitioners as a way to facilitate detailed, culturally responsive assessment of First Nations children’s knowledge and skills by local experts in their communities using local languages. If practitioners come from other cultural backgrounds to work with First Nations Australian families, this approach may be useful for application within intercultural relationships after careful discussion and consideration by local decision makers [64]. These methods could *only* be applied in meaningful, sustained and effective partnership with families and local community experts [59]. Measurements of child development and wellbeing can only be valid, meaningful and useful to communities if they are localised, contextualised, discussed, interpreted, elaborated, inquired into and acted upon by holders of local Indigenous Knowledges [34].

### The importance of using culturally matched child development frameworks

Yolŋu in this study placed a strong emphasis on *‘developing a strong Yolŋu identity’* and recognising a child’s strengths which may be “inside his bloodstream”. These priorities were reflected in what Yolŋu assessed with an emphasis on *gurruṯu* (kinship) concepts and connections, and the deepening knowledge, application and expression of identity and connections as children grow up and throughout Yolŋu life.

Yolŋu assessed children with consciousness of skills and knowledge passed on from generation to generation within a family, acceptance of individual differences in development, and awareness of the influences of a child’s family and community environment and experiences. These elements are assessed and considered holistically, not separately from, detailed observations of the individual child. Strengths may be interpreted as leadership potential. Challenges may be interpreted as areas where a child needs to be provided with more experience and encouragement. Assessments are made within a context of constant immersion in testing and teaching amongst many family members.

This article has explored some of the many ways Yolŋu assess Yolŋu children–for example: monitoring children’s awareness of appropriate and respectful behaviour and cultural conventions; analysing the skills underlying these behaviours (e.g. application of the *gurruṯu* (kinship) system; awareness of different Yolŋu languages and who the languages belong to); assessing the accuracy of children’s memory of knowledge that had been passed on to them; observing how children demonstrated knowledge of their connections through their confidence in their practice of Yolŋu skills (e.g. dancing at *buŋgul* (ceremony); hunting for shellfish); noticing what children notice in the environment around them. These are just some examples of “skills that Balanda don’t see”.

Some of these skills may overlap with categories and stages of development introduced from non-Yolŋu theories and frameworks of child development. However, to reinterpret these skills through non-Yolŋu frameworks risks losing depth and context and “dragging” Yolŋu children and families to a Balanda (non-Indigenous) world. For example, when Yolŋu children avoid saying words which sound like the name of someone who has passed away they demonstrate complex phonological awareness and flexible language skills; when a Yolŋu child gathers rock oysters with their family they demonstrate hand-eye coordination and fine motor skills. But these skills demonstrate much, much more than what can be seen through a non-Yolŋu lens. When Yolŋu and non-Yolŋu assessors both notice a child’s skills but interpret them differently, this can be an opportunity for discussion and for building shared understanding of the child. Development of such shared understanding requires assessors to have strong intercultural communication skills to effectively support Yolŋu aspirations for children to learn how to live well in the two systems that operate in Yolŋu communities (a Yolŋu system and a Balanda system). This aspiration is reflected in our project’s title: “*Ŋuthanmaram djamarrkuḻiny’ märrma’kurr romgurr—Growing up children in two worlds”* [9].

Yolŋu children’s skills are held and understood in very specific and important ways within Yolŋu frameworks. Respecting Yolŋu control over ways of understanding and assessing children must be foregrounded to uphold the rights of Indigenous peoples [1] and to support cultural maintenance of Yolŋu ways of growing up Yolŋu children as “people with brains, people with vision and dreams, we are people with dignity” (Jane Garrutju, [40]). To take knowledge of Yolŋu ways of assessing children and apply it without Yolŋu is unethical, disempowering and risks threatening the “cultural inheritance of past, current and future generations, and the links which bind the generations together” [65 p4].

### Limitations

Yolŋu are not one homogenous group of people but are a group of many clans and languages. This research has been conducted with a sample of families from one very remote community in North-East Arnhem Land. The findings have been shared as examples of how Yolŋu families recognise and understand their children’s development and learning. The research team acknowledges the importance and value of diversity within this remote community and across First Nations Australian communities. Ways of working together collaboratively and responsively, across linguistic and cultural differences, are complex and require ongoing research. Our findings, and the principles they demonstrate, may be useful for other families and people working with communities in other areas. However, it is imperative that each family is recognised and respected as experts in their own local cultural context. It is likely that each family will have their own strengths, priorities and methods of raising their children. This research may serve as a prompt for discussing family strengths and processes.

## Conclusion

Indigenous peoples have rights to culturally congruent assessment of their children. This can only be achieved when experienced assessors who share the child’s culture and language are recognised as experts. Yolŋu can see, hear and recognise how a Yolŋu child is responding to the many rich and complex layers in the teaching going on around them all the time. Yolŋu families are experts in understanding Yolŋu strengths, contexts and expectations: through close observation in everyday life, a child’s identity and learning is recognised and understood.

Non-Yolŋu assessment frameworks can *not* provide valid or holistic measures of a Yolŋu child’s development. Our findings demonstrate that Yolŋu have strong processes for assessing what they consider important in the development of their own children, nurturing their strengths and supporting their needs. Reconceptualising assessment requires non-Indigenous partners to recognise and respect First Nations processes and priorities, actively shifting control to First Nations Australian experts. Practitioners have a role in challenging culturally mismatched systems of assessing First Nations Australian children. Systemic change will require the support of policy which upholds the rights of Indigenous peoples to maintain their own knowledges and practices across generations.

## Supporting information

S1 File PLOS questionnaire: Inclusivity in global research. (DOCX)
